# Integrative transcriptome and proteome analyses of *Trichoderma longibrachiatum* LC and its cellulase hyper-producing mutants generated by heavy ion mutagenesis reveal the key genes involved in cellulolytic enzymes regulation

**DOI:** 10.1186/s13068-022-02161-7

**Published:** 2022-06-03

**Authors:** Miaoyin Dong, Shuyang Wang, Fuqiang Xu, Guoqing Xiao, Jin Bai, Junkai Wang, Xisi Sun

**Affiliations:** 1 Institute of Modern Physics, Chinese Academy of Sciences, 509 Nanchang Rd, Lanzhou, 730000 Gansu People’s Republic of China; 2 Institute of Biology, Gansu Academy of Sciences, 197 Dingxi South Rd, Lanzhou, 730000 Gansu People’s Republic of China; 3 University of Chinese Academy of Sciences, No.19A Yuquan Road, Beijing, 100049 People’s Republic of China; 4 College of Physics and Electronic Engineering, Northwest Normal University, 967Anning East Rd, Lanzhou, 730000 Gansu People’s Republic of China

**Keywords:** *Trichoderma longibrachiatum*, Cellulase, Hyper-producing mutants, Transcriptomics, Proteomics

## Abstract

**Background:**

The major challenge of facing the efficient utilization of biomass is the high cost of cellulolytic enzyme, while the *Trichoderma longibrachiatum* plays an essential role in the production of industrial enzymes and biomass recycling.

**Results:**

The cellulase hyper‑producing mutants of LC-M4 and LC-M16 derived from the wild type *T. longibrachiatum* LC strain through heavy ion mutagenesis exhibited the high-efficiency secretion ability of cellulase and hemicellulose. The FPase activities of LC-M4 (4.51 IU/mL) and LC-M16 (4.16 IU/mL) mutants increased by 46.91% and 35.5% when compared to the LC strain, respectively. Moreover, these two cellulase hyper-producing mutants showed faster growth rate on the cellulosic substrates (Avicel and CMC-Na) plate than that of LC strain. Therefore, an integrative transcriptome and proteome profiling analysis of *T. longibrachiatum* LC and its cellulase hyper‑producing mutant LC-M4 and LC-M16 were employed to reveal the key genes involved in cellulolytic enzymes regulation. It was showed that the transcriptome and proteome profiles changed dramatically between the wild strain and mutant strains. Notably, the overlapped genes obtained from integrative analysis identified that the protein processing in ER involved in protein secretory pathway, starch and sucrose metabolism pathway and N-glycan biosynthesis pathway were significantly changed both in cellulase hyper-producing mutants and thereby improving the enzyme secretion efficiency, which maybe the main reason of cellulase hyper-production in LC-M4 and LC-M16 mutants. In addition, the three DEGs/DEPs (PDI, Sec61, VIP36) related with protein secretion in ER and two DEGs/DEPs (OST, MOGS) related with N-glycan biosynthesis were identified as key candidate genes participating in enzyme protein biosynthesis and secretion.

**Conclusions:**

In this study, a hypothetical secretory model of cellulase protein in filamentous fungi was established on the basis of DEGs/DEPs and key genes identified from the omics analysis, which were of great guidance on the rational genetic engineering and/or breeding of filamentous fungi for improving cellulase production.

**Supplementary Information:**

The online version contains supplementary material available at 10.1186/s13068-022-02161-7.

## Background

Lignocellulosic biomass, including agricultural residues, forestry wastes, woody biomass, energy crops, were widely regarded as a potential feedstock for the production of biofuels and other value-added products through integrated bio-refineries, including pretreatment, enzyme hydrolysis, and microbial fermentation [1, 2]. For liquid fuels production, the lignocellulose was needed to be firstly hydrolyzed to fermentable sugars and then fermented by microorganisms [3]. Of these, the low hydrolysis efficiency and production yields, high costs of cellulolytic enzymes limited the industrial development of bio-refineries from cellulosic biomass [4].

Filamentous fungi, especially *Trichoderma *spp. are well-known efficient producers of cellulolytic enzymes for lignocellulose bio-refinery [5]. While the native extracellular enzyme system secreted from fungus cannot meet the needs of industrial application on the large scale. Therefore, it is imperative to enhance the enzyme production and cellulose hydrolysis of *T. longibrachiatum* by the strain improvement through random mutagenesis technology [6]. To date, the heavy ion irradiation has been successfully used for mutation breeding of microorganisms to obtain novel strains with industrial application potential and produced a large number of excellent mutants [7–9] due to its high linear energy transfer (LET), high relative biological effectiveness (RBE), and broad mutation spectrum [10]. Heavy ion irradiation could induce a substantial DNA damage because there is a failure to repair correctly or is unrepairable [2], thereby producing numerous mutants.

Although there are many studies focused on the signaling cascades and relevant transcriptional regulatory networks involved in cellulolytic enzymes synthesis and protein secretion in filamentous fungi [11–17], the molecular mechanisms regulating cellulase and hemicellulose genes expression remain elusive due to the complexity of regulatory system [18]. Thus, the identification of more-specific regulatory genes or regulators involved in cellulolytic enzyme genes expression has great potential for engineering strains and further enhancing enzyme production. The recent development in next-generation sequencing technologies, a powerful tool for measuring global genes and protein expression, has significantly enhanced our understanding for the discovery of novel genes and its functions involved in specific biological processes [19]. Thus, a coalition analysis of the transcriptome and proteome between wild-type strain and its cellulase hyper-producing mutants can provide the key insights into the cellulolytic enzyme synthesis regulation and protein secretion in filamentous fungi from the mRNA and protein levels.

Therefore, in this study, the integrative analysis of transcriptome and label-free quantitative proteome of *T. longibrachiatum* LC and its cellulase hyper-producing mutants were carried out to identify the candidate genes that regulated the cellulolytic enzymes synthesis and secretion process. In addition, the omics data obtained from this study could provide the significant insights into omics resources and may help further research on the rational genetic engineering of *T. longibrachiatum* to enhance cellulolytic enzyme productivity.

## Materials and methods

### Strains and culture condition

*T. longibrachiatum* LC-M4 [20, 21] and LC-M16 strains, cellulase hyper-producing mutant, were generated by the ^12^C^6+^ ion irradiation mutagenesis from the wild type of *T. longibrachiatum* LC strain (NCBI Accession No.MW193401) isolated from agricultural waste. Screening of mutant strains after mutagenesis were performed as described by our previous study [2]. These strains were grown on potato dextrose agar (PDA) slant and stored in the Biophysics Laboratory of Institute of Modern Physics, Chinese Academy of Sciences (CAS).

The preculture medium was a modified Mendel medium containing 5 g of peptone, 10 g of glucose, 1.4 g of (NH_4_)_2_SO_4_, 2.0 g of K_2_HPO_4_, 0.3 g of CaCl_2_, 0.3 g of MgSO_4_, 0.3 g of urea, 2 mL of Tween-80, 0.0016 g of MnSO_4_·H_2_O, 0.005 g of FeSO_4_·7H_2_O, 0.002 g of CoCl_2_, 0.0014 g of ZnSO_4_·7H_2_O per liter of deionized water. The fermentation medium was the same as preculture medium except that 17 g of corn syrup, 20 g of Avicel and 10 g of wheat bran were used instead of 5 g/L peptone and 10 g/L glucose.

### Phenotypic analysis

For comparing the growth characteristics of *T. longibrachiatum* LC and its mutants on plates, the media with different carbon sources of 20 g/L lactose, 20 g/L soluble starch, 20 g/L glucose, 20 g/L sucrose, 20 g/L Avicel and 20 g/L CMC-Na and MM solution [22] were prepared, respectively. 1 µL of spore suspension (1 × 10^6^ spores/mL) of various strains was inoculated onto the plates and incubated at 30 °C. Then, the colony diameter of strain grown on plate was analyzed after 3 days of incubation.

### Preparation of supernatant and mycelia

2 mL of spore suspension (1 × 10^6^ spores/mL) of *T. longibrachiatum* LC and its cellulase hyper-producing mutants were used to inoculate 50 mL of preculture medium for 24 h cultivation at 30 ℃ and 200 rpm. The seed was inoculated at 5% (v/v) into 50 ml fermentation medium at the same condition and the mycelium was collected after 48 h, 72 h, 96 h, 120 h and 144 h of fermentation, respectively, and the supernatants after 192 h of fermentation were used for proteome analysis and enzyme activity assays. At last, the mycelia samples of each time points from LC, LC-M4 and LC-M16 strain were pooled and the total nine samples were prepared for the next-generation sequencing [23]. All experiments were performed in three biological replicates.

### Enzyme activity and soluble protein concentration assays

The enzyme activities of FPase, CMCase, β-glucosidase, pNPCase, xylanase were determined as described by our previous study [21]. Briefly, the FPase activities were assayed by incubating filter paper (50 mg, 1.0 × 6.0 cm) in a reaction mixture consisting of citric acid buffer (1.5 mL, 0.05 M, pH 4.8) and diluted crude enzyme (0.5 mL) for 60 min at 50 °C. While the CMCase and β-glucosidase activities were determined by incubating 1.5 mL of 1% CMC-Na and 1% salicin (dissolved in citric acid buffer (0.05 M, pH 4.8)) as substrate and 0.5 mL of diluted crude enzyme for 30 min at 50 °C, respectively. Xylanase activities were investigated by incubating 1 mL of substrate (1% xylan (from beechwood, yuanye, China)) and 1 mL of diluted crude enzyme for 30 min at 50 °C. The concentrations of reducing sugar were determined by using the DNS method [24] and enzyme activity unit (IU) was defined as the amount of enzyme that released 1 μmol of reducing sugars per minute under assay conditions. pNPCase activities were determined using a reaction mixture consisting of 150 μl of substrate (1 mg/mL p-nitrophenyl-β-d-cellobioside (pNPC)) and 50 μl of diluted crude enzyme and incubated at 50 °C for 30 min. Then, the reaction was stopped by adding 150 μl of 10% (w/v) Na_2_CO_3_ solution and the absorbance was determined at 405 nm. The enzyme activity unit (IU) was defined as the amount of enzyme that released 1 μmol of p-nitrophenol per minute under assay conditions [25]. The soluble protein concentrations in supernatant after 192 h of fermentation were determined by using the Bradford protein assay kit (Solarbio Biotech, Beijing, China) according to the manufacturer’s protocol [16].

### RNA sequencing and data analysis

Total RNA used for RNA-seq assays were extracted from the frozen mycelia samples using TRIzol^®^ RNA kit according to its manufacturer’s instructions. The RNA integrity and quality were analyzed by using the 2100 RNA Nano 6000 Assay Kit (Agilent Technologies, CA, USA) and Nanodrop spectrophotometer system, respectively. The cDNA libraries for sequencing were purified through the AMPure XP system and prepared by PCR amplification, then the library quality was examined by using the Agilent 2100 system. Finally, the library sequencing was performed with an Illumina HiSeq2500 instrument by Shanghai Applied Protein Technology (Shanghai, China). The clean reads were aligned and mapped onto the reference genome of *T. longibrachiatum* ATCC 18,648 (https://www.ncbi.nlm.nih.gov/genome/18220?geno-me_assembly_id=370100) by using Hisat2 software. For gene expression analysis, the number of reads corresponding to each gene was calculated by FeatureCounts software and the FPKM (fragments per kilobase per million) of each gene was calculated on account of the length of the gene and read counts mapped to this gene. Genes with an adjusted P value of less than 0.05 and fold-change of more than 2 were deemed to be differentially expressed between the two samples and the differentially expressed genes (DEGs) were identified by using the DESeq2 (Anders et al., 201x) R package software.

### Quantitative real-time PCR analysis

Totally, 20 genes were selected to evaluate the validity of RNA-seq data by real-time PCR. The primers were designed using OligoArchitect™ Online system (http://www.oligoarchitect.com/AlternatePrimers.jsp) and listed in Additional file 1: Table S1. In this study, the *sar* gene was selected as the control gene for normalization as previously reported [26, 27]. Quantitative real-time PCR experiments were performed on Applied Biosystems QuantStudio 5 (Thermo Fisher, USA) with SYBR Green PCR master mix (Novogene, China). The PCR reactions were performed in triplicate, in which the 2^−ΔΔCT^ method was used for calculating the fold-change of target genes.

### Protein extraction and digestion

Proteins were extracted from the supernatants after 192 h of fermentation by using the SDT (4% (w/v) SDS, 100 mM Tris–HCl pH 7.6, and 0.1 M DTT) lysis method described by Zhu et al. [28] and quantified using the BCA Protein Assay Kit. Then, the proteins were digested by filter-aided sample preparation (FASP) digestion into peptides according to the Wisniewski et al. [29]. Briefly, the extracted proteins were dissolved in 8 M urea, then the dithiothreitol (DTT) and chloroacetamide were added for 30 min incubation in the dark at room temperature. Subsequently, the proteins were digested with trypsin (Promega, USA) in TEAB buffer overnight at 37 ℃. Finally, the peptides were desalted by C18 Cartridge and concentrated by vacuum centrifugation.

### LC–MS/MS analysis

The peptides were reconstituted with 40 µL 0.1% (v/v) formic acid and the LC–MS/MS analysis were carried out by the ultra-high resolution Q-Exactive mass spectrometer (Thermo Scientific) coupled with an Easy nLC (Thermo Fisher Scientific). The peptides were loaded onto the trap column (Thermo Scientific Acclaim PepMap100, 100 μm × 2 cm, nanoViper C18) and then separated at a flow rate of 300 nL/min by an analysis column (Thermo scientific EASY column, 10 cm, ID75μm, 3 μm, C18-A2). MS analysis with a survey scan (300–1800 m/z) was performed in the positive ion mode at a resolution of 70,000 at 200 m/z, maximum inject time of 50 ms, automatic gain control target of 1 × 10^6^ and dynamic exclusion of 60.0 s. In addition, the data of peptides and peptide fragments were acquired as follows: 20 fragment files collected after full scan, higher-energy collision dissociation (HCD) fragmentation, 2 m/z of isolation window, 17,500 resolutions (at 200 m/z) of HCD spectra, 30 eV of normalized collision energy and 0.1% of the underfll ratio. The MS raw data were further analyzed by MaxQuant (v1.5.3.17).

Proteins with the P value of less than 0.05 and fold-change of more than 2 were defined as differently expressed proteins (DEPs). The functional annotation of DEPs were performed by Gene Ontology (GO) and the Kyoto Encyclopedia of Genes and Genomes (KEGG) pathways databases using Blast2Go (https://www.blast2go.com/) [30], and its richness analysis were carried out by Fisher test.

## Results

### Comparison of growth phenotype and enzyme activities between *T. longibrachiatum* LC and its mutants

**Fig.1:**
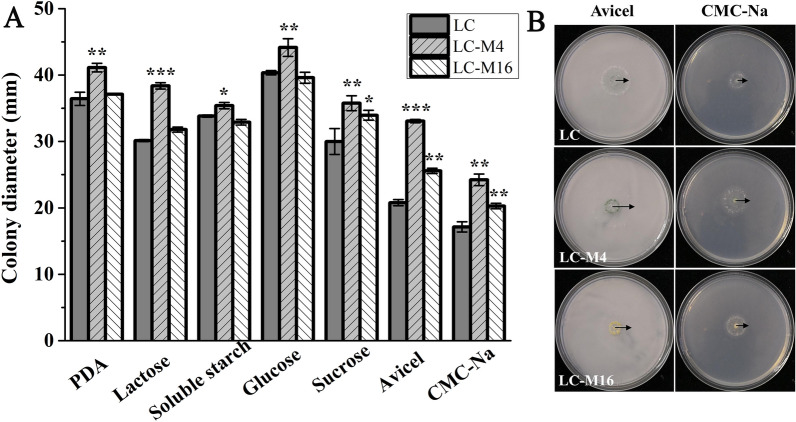
Growth of *T. longibrachiatum* LC and its cellulase hyper-producing mutants (LC-M4 and LC-M16) on agar plates after 3 days of incubation. **A** Comparison of colony diameters between *T. longibrachiatum* LC, LC-M4 and LC-M16; **B** photographs of *T. longibrachiatum* colonies on avicel and CMC-Na plates. *p < 0.05; **p < 0.01; ***p < 0.001. Error bar standard deviation of three replicates

The enzyme hyper-producing mutant of LC-M4 and LC-M16 strains generated by heavy ion mutagenesis were derived from the wild-type strain of *T. longibrachiatum* LC (NCBI Accession No. MW193401) isolated from the agricultural waste. The growth characteristics of wild-type strain of *T. longibrachiatum* LC and its cellulase hyper-producing mutants of LC-M4 and LC-M16 on lactose, soluble starch, glucose, sucrose, Avicel and CMC-Na and PDA plates are presented in Fig. 1. It was noted that the LC-M4 strain showed the largest colonies among three strains on all plates with various carbon sources after 3 days of growth. The LC-M16 strain also showed larger colonies on the sucrose, Avicel and CMC-Na plates when compared to the wild-type strain of *T. longibrachiatum* LC. The cellulase hyper-producing mutants of LC-M4 and LC-M16 strains grown on the Avicel and CMC-Na plates showed a larger colony than that of LC strain. In addition, it was observed that the spore color of LC-M16 strain was yellow, whereas the LC and LC-M4 strain were dark green (Fig. 1B).

**Fig.2:**
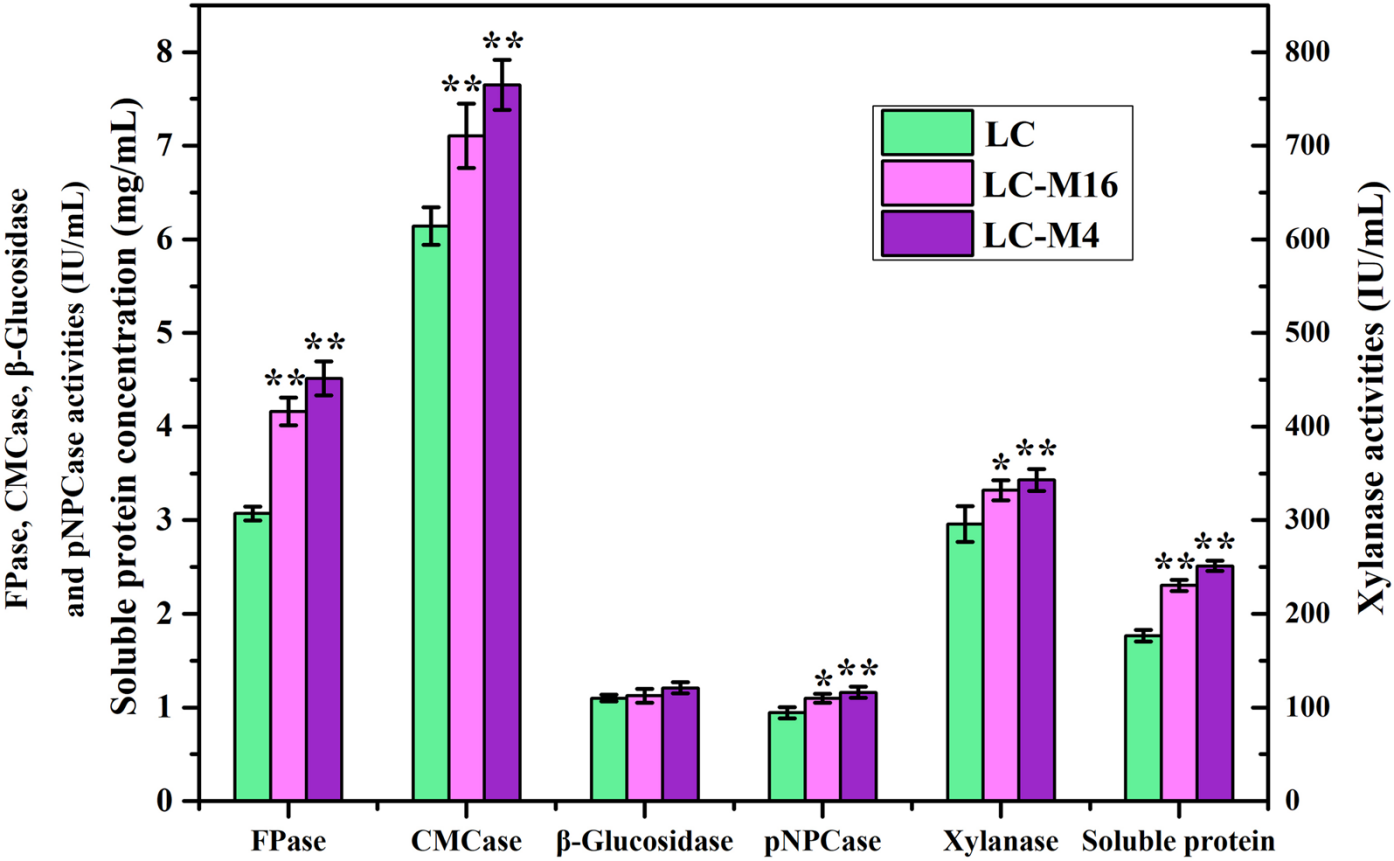
Enzyme activities of FPase, CMCase, β-glucosidase, pNPCase, Xylanase and soluble protein concentration in supernatant after 192 h of fermentation by the wild-type strains of *T. longibrachiatum* LC and its mutants of LC-M4 and LC-M16. *p < 0.05; **p < 0.01. Error bar standard deviation of three replicates

In the present study, the FPase, CMCase, β-glucosidase, pNPCase and xylanase activities were determined in order to comprehensively characterize cellulolytic enzymes produced by enzyme hyper-producing mutants. The results showed that the FPase and CMCase activities from two mutants were significantly higher (*p* < 0.01) than that of the wild strain of LC (Fig. 2). Especially, the FPase activities of LC-M4 (4.51 IU/mL) and LC- M16 (4.16 IU/mL) mutants increased by 46.91% and 35.5% when compared to the wild-type strain, respectively. LC-M4 (*p* < 0.01) and LC-M16 (*p* < 0.05) mutants exhibited higher pNPCase and xylanase activities when compared to the LC strain, whereas there were no significant difference of β-glucosidase activities between two mutants and wild strain, which indicated that the traits of high enzyme activity in mutants were mainly attributed to the high CMCase, pNPCase and xylanase activities. Similarly, a higher soluble protein concentration in *T. longibrachiatum* mutants of LC-M4 and LC-M16 were observed after 192 h of fermentation and reached 2.51 mg/mL and 2.31 mg/mL when compared to the wild-type strains of LC (*p* < 0.01), respectively. Therefore, we can speculate that the increase of secreted proteins was the main reason of enhancing enzyme activities in mutants.

### Transcriptomic analysis of *T. longibrachiatum* LC, LC-M4 and LC-M16

To further reveal the molecular mechanism of high-yield cellulolytic enzymes production in the mutant LC-M4 and LC-M16 and screen candidate genes of regulating cellulase and/or xylanase gene expression, firstly, a comparative transcriptomics analysis between wild type and mutant strains were performed. In total, approximately 6.51G, 6.36G and 6.32G clean bases with 93.4%, 93.1% and 93.1% Q30 bases were obtained from the LC, LC-M4 and LC-M16 libraries, respectively (Additional file 2: Table S2). The clean reads representing an average 89.45-fold coverage of the *T. longibrachiatum* ATCC_18648 genome. As a result, a total of 11,616 genes were detected in all libraries, among which 2620 (1133 up- and 1487 down-regulated) and 2709 (1370 up- and 1339 down-regulated) were identified as differently expressed genes (DEGs) in LC-M4 vs LC and LC-M16 vs LC groups, respectively (Table 1, Additional file 3: Fig S1, Additional file 4: Table S3, Additional file 5: Table S4).

**Table 1:** Summary of genes and proteins identified from the RNA-Seq and MS data

Terms	Transcriptome		Proteome	
	LC-M4_ LC	LC-M16_ LC	LC-M4_ LC	LC-M16_ LC
Total genes/proteins	11616	11616	1374	1374
DEGs/DEPs	2620	2709	330	338
Up-regulated	1133	1370	202	188
Down-regulated	1487	1339	128	150
Co-expressed genes/proteins	1100	1159	1100	1159
Co-expressed DEGs-DEPs	127	140	127	140

**Fig.3:**
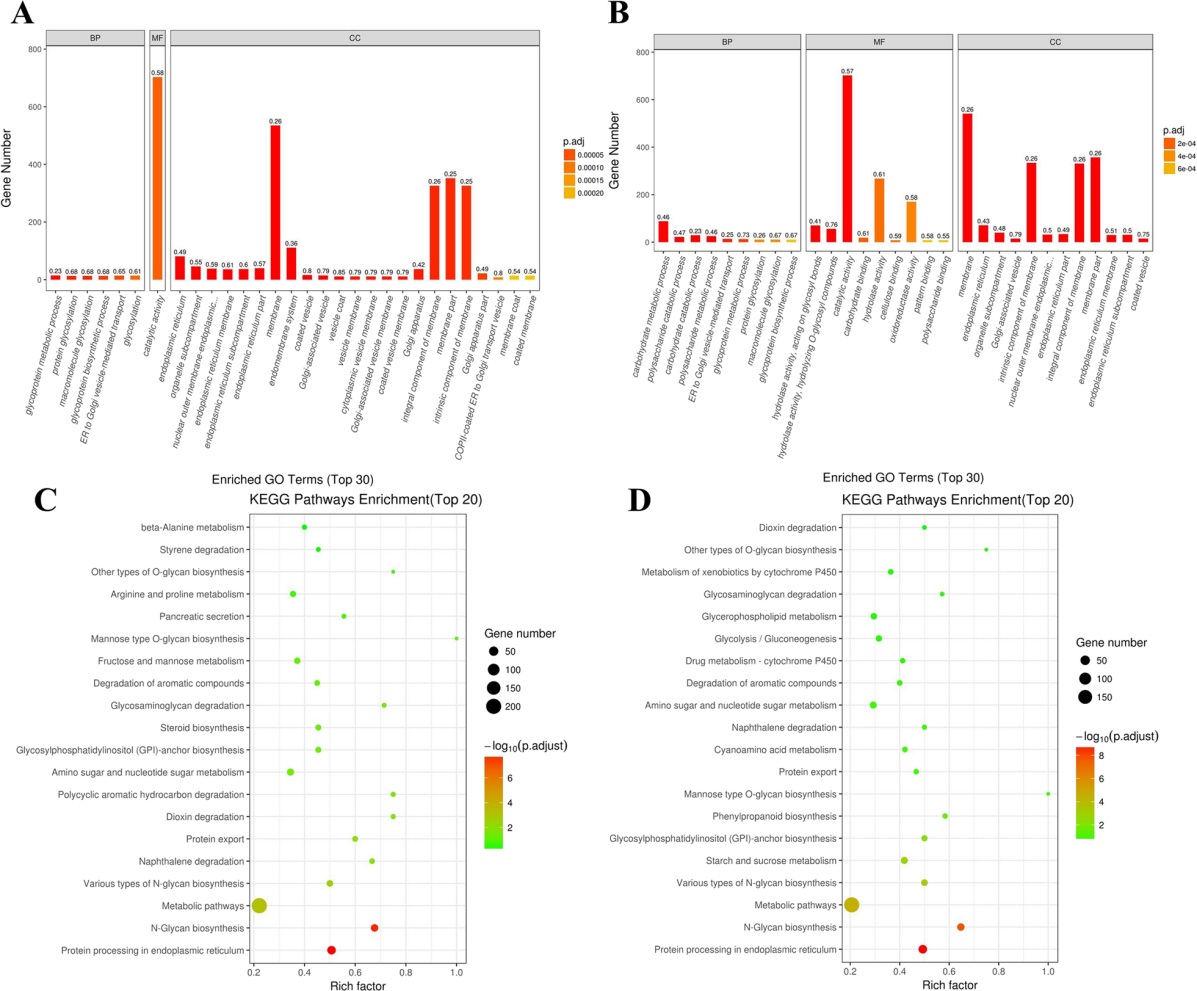
Functional analysis of differentially expressed genes using GO and KEGG pathways in the *T. longibrachiatum* wild type and mutant strains. **A**, **B** The analysis results of GO of LC-M4_LC and LC-M16_LC, respectively; **C**, **D** the analysis results of KEGG of LC-M4_LC and LC-M16_LC, respectively

The enrichment analysis of GO terms indicated that the most DEGs were involved in glycoprotein metabolic process and carbohydrate metabolic process in the biological process (BP) category in LC-M4_LC and LC-M16_LC groups, respectively. While the catalytic activity comprised the highest proportions of DEGs in the molecular function (MF) in both LC-M4_LC and LC-M16_LC groups. In cell components (CC) categories, the most significant terms were endoplasmic reticulum and membrane in the LC-M4_LC group and membrane and intrinsic component of membrane in the LC-M16_LC group (Fig. 3a, b). Subsequently, we performed the KEGG enrichment analysis of DEGs to reveal the transcriptional change in metabolic pathways among the wild type and mutant strains. The most DEGs were significantly enriched in protein processing in endoplasmic reticulum, N-glycan biosynthesis, metabolic pathways and protein export in both LC-M4_LC and LC-M16_LC groups (Fig. 3c, d). Among them, the main cellulase genes of *cbh1, cbh2, eg1* and *eg2* in the starch and sucrose metabolic pathways in mutants were 2–5 times higher than in wild strains, and the other cellulase enzymes genes of *bgl, bgl2* and *egIII* were also significantly increased in mutant strains. In addition, the transcription activator of *xyr1* was up-regulated both in mutants, which indicated that the significant up-regulation of main enzyme genes was closely related to the positive regulation of *xyr1* in mutant strains (Table 2, Additional file 4: Table S3, Additional file 5: Table S4). Additionally, the relatively expression level of selected DEGs were tested by using quantitative real-time PCR (RT-qPCR). In total, 20 DEGs associated with the enzyme secretion and cell growth in fungi were selected and performed the expression level verification. The coefficient of transcriptome and RT-qPCR results were 0.83, which indicated that the reliability and validity of RNA-seq data obtained from the *T. longibrachiatum* LC and its cellulase hyper-producing mutants (Additional file 6: Fig S2).

**Table 2:** Summary of transcription factors up/down-regulated in *T. longibrachiatum* mutants of LC-M4 and LC-M16 vs LC

Gene_ID	log_2_FC	Padj	Gene description
LC-M4 vs LC			
M440DRAFT_1394263	0.9418	1.36E–16	Xylanase regulator 1
M440DRAFT_1389588	− 1.1014	5.09E–12	Fungal-specific transcription factor domain-containing protein
M440DRAFT_1389710	− 1.8322	0.022146	Basic leucine zipper transcription factor domain-containing protein
M440DRAFT_1427075	− 2.5259	2.26E–89	Dolichol phosphate-mannose biosynthesis regulatory
M440DRAFT_1441016	3.7983	1.10E–77	N-terminal fungal transcription regulatory domain-containing protein
M440DRAFT_69088	− 1.1739	4.88E–24	Protein phosphatase 2A regulatory B subunit
M440DRAFT_1327308	− 1.0717	1.36E–12	G-protein signaling regulator protein
LC-M16 vs LC			
M440DRAFT_1394263	1.4284	5.68E–23	Xylanase regulator 1
M440DRAFT_1441016	2.9991	2.46E–63	N-terminal fungal transcription regulatory domain-containing protein
M440DRAFT_1327308	− 1.1587	5.54E–16	G-protein signaling regulator protein
M440DRAFT_1427075	− 1.9314	3.61E–36	Dolichol phosphate-mannose biosynthesis regulatory
M440DRAFT_1199307	1.0783	1.05E–11	Transcription factor-like protein
M440DRAFT_1441655	− 1.2298	9.63E–10	Basic-leucine zipper transcription factor

### Comparison of proteome of *T. longibrachiatum* strains

Label-free quantitative proteomics technology was used to obtain the complete proteome of *T. longibrachiatum* strains grown in wheat bran and Avicel as the carbon source. A total of 9162 peptides and 1374 proteins were identified in the proteome of LC, LC-M4 and LC-M16. Among these, 330 (202 up- and 128 down-regulated) and 338 (188 up- and 150 down-regulated) were identified as differentially expressed proteins (DEPs) in the LC-M4_LC and LC-M16_LC groups, respectively (Table 1). However, due to the novelty of the enzyme production microorganism, many detected findings were identified as hypothetical proteins.

**Fig.4:**
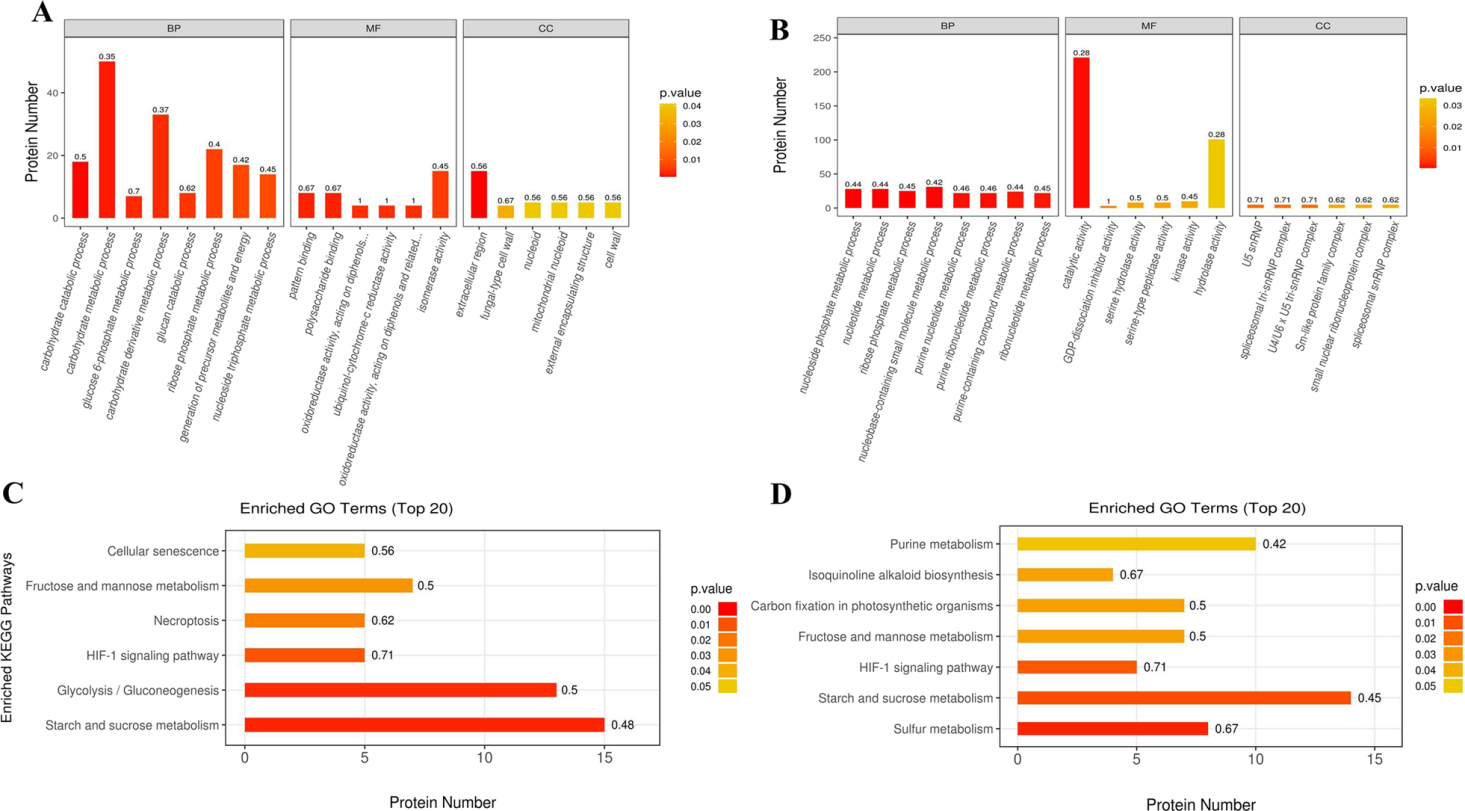
Functional annotations of differentially expressed proteins using GO and KEGG pathways in the *T. longibrachiatum* wild type and mutant strains. **A**, **B** The analysis results of GO of LC-M4_LC and LC-M16_LC, respectively; **C**, **D** the analysis results of KEGG of LC-M4_LC and LC-M16_LC, respectively

The results of GO enrichment analysis indicated that in the BP category, the DEPs were enriched in carbohydrate catabolic process in the LC-M4_LC group and in nucleoside phosphate metabolic process in the LC-M16_LC group, respectively. In the MF category, the highest proportions of DEPs were involved in polysaccharide binding and isomerase activity in LC-M4_LC group and catalytic activity in LC-M16_LC group, respectively. The extracellular region in the LC-M4_LC group and spliceosomal tri-snRNP complex in the LC-M16_LC group showed the significant enrichment of DEPs in the CC category (Fig. 4a, b). In addition, the DEPs in the LC-M4_LC group were enriched in starch and sucrose metabolism, glycolysis/gluconeogenesis and fructose and mannose metabolism. While the DEPs in the LC-M16_LC group were enriched in sulfur metabolism, starch and sucrose metabolism, isoquinoline alkaloid biosynthesis and purine metabolism (Fig. 4c, d). Interestingly, the main cellulase protein of CBHI, CHBII, EGII and EGIII in starch and sucrose metabolism pathway in LC-M4 mutant were 2.9-fold, 5.8-fold, 6.2-fold and 2.4-fold higher than that of the LC strain, respectively. In addition, the expression of alpha-amylase in LC-M4 mutant were also 3.2-fold higher when compared to the wild type of LC strain (Additional file 7: Table S5, Additional file 8: Table S6).

### Coalition analysis of transcriptome and proteome of *T. longibrachiatum* strains

**Fig. 5:**
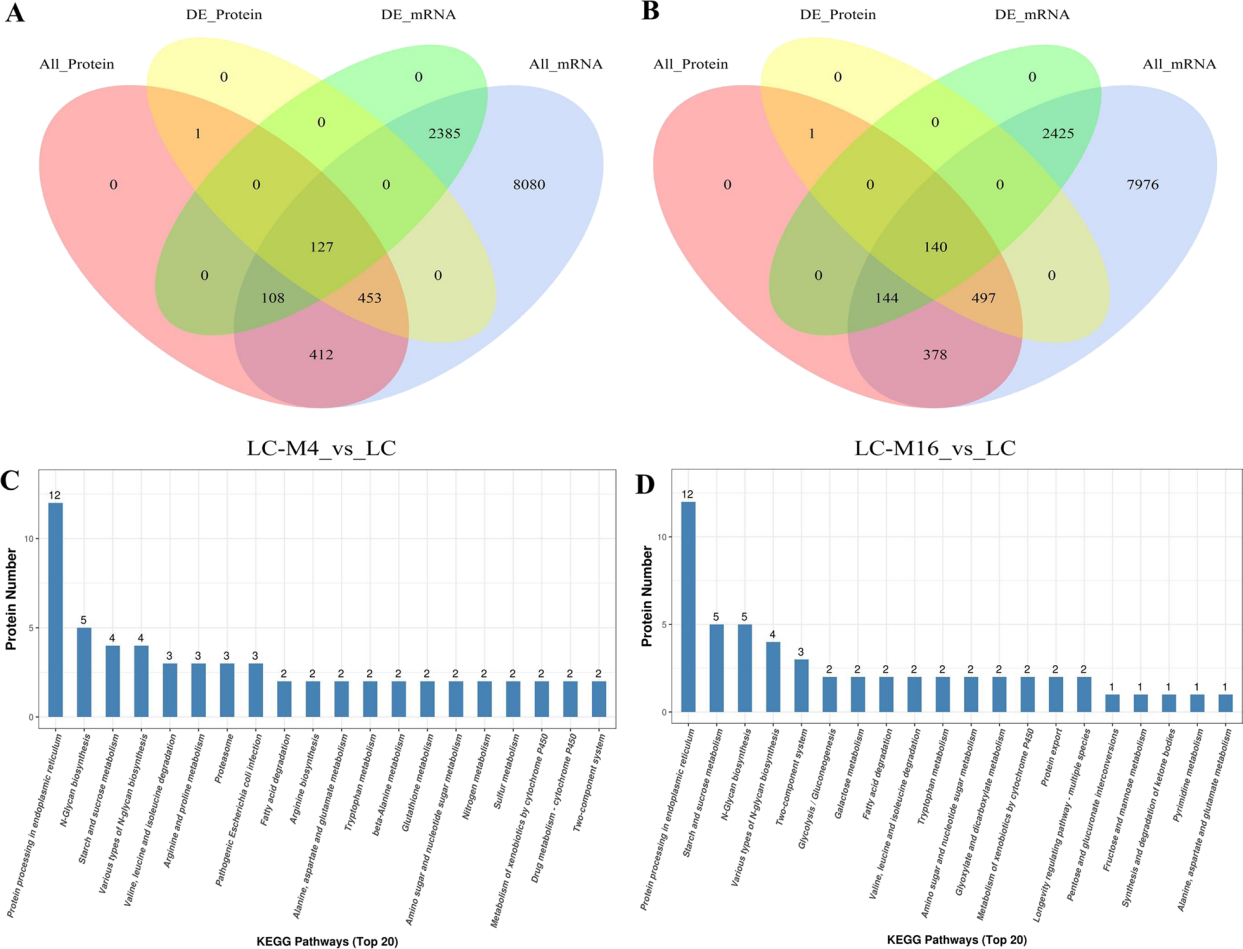
The correlations analysis of RNA-Seq and proteins in *T. longibrachiatum* strains. Venn diagram of all detected mRNA and proteins in LC-M4_LC (**A**) and LC-M16_LC (**B**), respectively. KEGG pathway annotation analysis of co-regulated DEGs/DEPs in LC-M4_LC (**C**) and LC-M16_LC (**D**), respectively

To obtain a global understanding of metabolic differences at the transcriptional and translational level between the *T. longibrachiatum* wild type and mutant strains, a coalition analysis of the transcriptome and proteome was performed. There were 1100 proteins were associated with the identified genes, but only 127 DEPs showed the corresponding changes at the transcriptional level in the LC-M4_LC groups (Fig. 5A, Additional file 7: Table S5). While 1159 proteins were associated with the detected genes in the LC-M16_LC groups, among which only 140 genes/proteins with common changes were detected and 497 genes showed no changes in transcript levels (Fig. 5B, Additional file 8: Table S6). Consistent with several previous studies on the mismatch between transcriptomes and proteome [31], our proteome has a weak correlation (*R* = 0.0952 and 0.0093) with the transcriptome in the LC-M4 and LC-M16 strains compared with the WT, respectively. The low expression correlation between mRNAs and proteins indicated that the proteins secreted by *T. longibrachiatum* might be influenced by a post-transcriptional and post-translational modifications. In addition, a previous study reported that the mRNAs are less stable and about 900 times less expression than proteins and existed with a higher dynamic range [32], which might be also explained the weak associations obtained in the present study. While a higher correlation (*r* = 0.761 and 0.7622) was identified with the same trend for common DEGs and DEPs in the LC-M4 and LC-M16 strains compared with the WT, respectively. Given that above 50% of genes in the *T. longibrachiatum* genome are unknown function, the DEGs/DEPs identified from the mutants will aid in revealing novel genes function in enzyme proteins secretory pathway [33].

### Analysis of potential metabolic pathways associated with the cellulolytic enzymes synthesis and secretion in *T. longibrachiatum*

The KEGG functional annotation analysis of co-regulated DEGs/DEPs in LC-M4_LC showed that the most DEGs/DEPs were involved in protein processing in ER, N-glycan biosynthesis, starch and sucrose metabolism, proteasome and nitrogen metabolism (Fig. 5C). Most were involved in protein processing in ER, starch and sucrose metabolism, N-glycan biosynthesis, two-component system and protein export in the LC-M16 strain compared with the WT (Fig. 5D). Comparative analysis indicated that the protein processing in ER (PDI, M440DRAFT_1464388; VIP36, M440DRAFT_1396316), starch and sucrose metabolism and N-glycan biosynthesis pathways (OST, M440DRAFT_359740; MOGS, M440DRAFT_1329096) were shared both in the two cellulase hyper-producing mutants compared with the wild-type strain. Therefore, we concluded that these above candidate pathways comment shared by DEGs and DEPs might have important roles in cellulase enzyme synthesis and section in *T. longibrachiatum.*

**Fig. 6:**
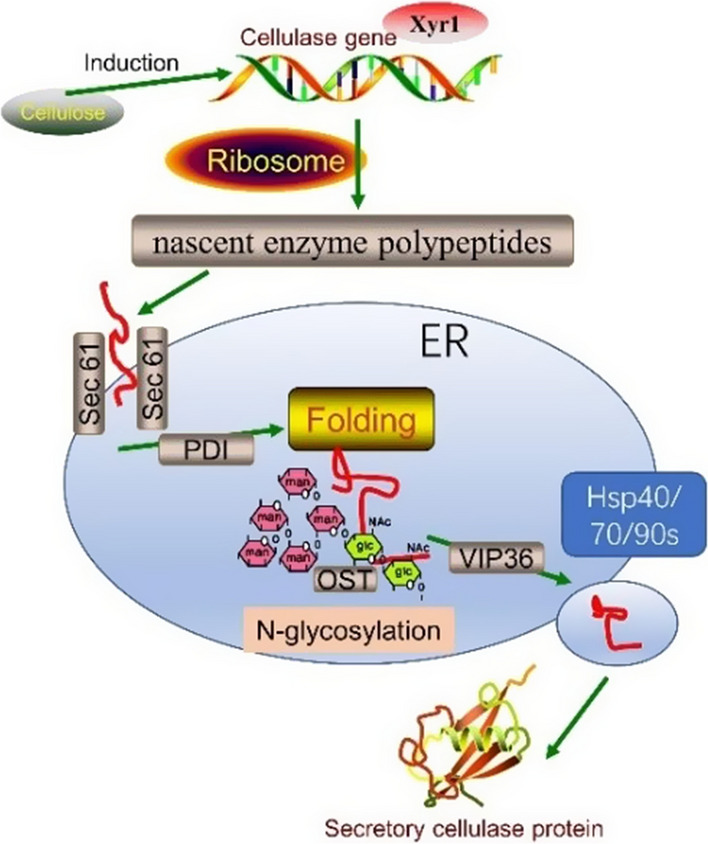
The hypothetical secretory model of cellulase protein in filamentous fungi

Based on the above results, a hypothetical secretory model of cellulase protein in filamentous fungi was established on the basis of DEGs/DEPs and key genes identified from the cellulase hyper‑producing mutants (Fig. 6). The main cellulase genes started to transcribe under the induction of cellulosic substrates, while being positively regulated by Xyr1, and translate into the nascent enzyme polypeptides on the ribosome. Then, the nascent polypeptides were transported to ER through the channel mediated by the Sec61 for folding and modification, including the conformation folding (PDI) and N-linked glycosylation modification (OST). Lastly, the mature enzyme protein was secreted to the cell exterior via the vesicles with the mediating of molecular chaperone associated with the protein secretory pathway. Therefore, this secretory model of cellulase were of great guidance on the rational genetic engineering and/or breeding of filamentous fungi via the overexpressing of key ER chaperones and folding enzymes as the targets.

## Discussion

The cellulase hyper-producing mutant of LC-M4 and LC-M16 strains were obtained from the wild-type strains of *T. longibrachiatum* LC after heavy ion mutagenesis. In addition, the growth phenotype of LC-M4 and LC-M16 mutant strains grown on the cellulosic substrate plates (Avicel and CMC-Na as the carbon source) showed the larger colonies than that of the wild-type LC strain after 3 days of incubation (Fig. 1), which indicated that the mutants had a faster growth rate on the cellulosic carbon source. In this study, the conidia of LC-M16 mutant are yellow, whereas its wild-type strain of LC were dark green. These phenotypic variation in mutants were mainly caused by the change of secondary metabolism induced by mutagenesis process [34]. Therefore, the mechanism of conidia color turning yellow in LC-M16 mutant requires further investigation.

The cellulase hyper-producing mutant of LC-M4 and LC-M16 strains exhibited the high-efficiency secretion ability of cellulase and hemicellulose (Fig. 2), which were consistent with the up-regulated main enzyme genes/proteins (CBH I, CBH II, EG2 and EG3) in mutants. In our previous study, one *A. fumigatus* mutant MS160.53 was derived from the wild strain MS13.1 after heavy ion mutagenesis, and the FPase activities (1.81 IU/ml) of mutant increased by 40.3% compared with the wild strain (1.29 IU/mL) [2]. For improving the cellulase production, the several excellent strain lineages of *T. reesei* (such as QM9414, RUT-C30) have been produced by various mutagenesis programs from the wild-type strain of QM6a [35, 36]. In addition, a mutant of *P. oxalicum* EU2106 with high-yield cellulase production was obtained from the wild type *P. oxalicum* HP7-1 after three rounds of Co^60^ γ-ray irradiation and two rounds of EMS/UV combined mutagenesis, and the FPase activities in mutant increased by 55.3% compared with the wild-type strains [12, 37]. Thus, these studies demonstrated that the random mutagenesis is one of the most effective way to enhance enzyme production in filamentous fungi.

Nevertheless, the genetic causes leading to cellulase overproduction remain relatively uncharacterized. For a long time, the exact identification of the genetic changes causing cellulase hyper-production have been gradually performed by laborious complementation [38], but its results remain limited due to the complexity of regulation system of enzyme secretion. In this study, the hyper-producing strains generated by random mutagenesis of heavy ion might contain multiple mutations in genome [39]. Therefore, we integrated the transcriptome and proteome analysis of wild type and mutant strains in cellulase inducing conditions to reveal the key candidate genes involved in cellulolytic enzymes regulation in *T. longibrachiatum.*

According to the integrative analysis of proteomics and transcriptomics, we identified important secreted CAZymes like two cellobiohydrolase (CBH I and CBH II), two endoglucanases (EG2 and EG3), three β-glucosidase (BGL1, cel3b and cel3c), and two xylanases, which were upregulated in *T. longibrachiatum* mutants at least once in all two omics except for β-glucosidase (cel3b). These results indicated that the post-transcriptional and post-translational regulation events might play more important roles in cellulolytic enzymes synthesis in LC-M4 than LC-M16 strain [40]. Zhao et al. [12] investigated the genomic, transcriptomic and secretomic profiling of *P. oxalicum* HP7-1 and its cellulase and xylanase hyper-producing mutant EU2106. They demonstrated that although the genes of the major cellulase and hemicellulase enzymes were significantly up-regulated in the mutant strain, the mutations located in catalytic residues of enzyme in EU2016 were not found.

Among the proteins secretory pathway leading nascent protein synthesized in ribosome to the cell exterior, the proteins folding in ER is one of the most important process, which needs a series of molecular chaperones and folding enzymes to promote the secreted proteins into their finally correct conformation [41]. Proteomics results showed that several key proteins involved in protein secretory pathway were significantly up-regulated in mutant strains. The molecular chaperones proteins involved in ER such as Hsp40s (M440DRAFT_1396706), Hsp70s (M440DRAFT_1441140), Hsp90s (M440DRAFT_1394770) were remarkably upregulated both in LC-M4 and LC-M16 strains, which were necessary to prevent protein aggregates of misfolded/unfolded and promote protein folding [42]. In addition, the cytosolic Hsp70s chaperone could interact with the nascent enzyme proteins and help to keep it in an unfolded conformation. Then the complex is translocated into the ER with the assistance of the chaperone BiP and membrane protein Sec63p.

The expression levels of the secretory pathway-related genes, Sec61(M440DRAFT_1404259), Sec62, Sec63, Sec23/24 were also significantly up-regulated in two mutants. Of these, the Sec61 forms a channel across the ER membrane and mediates the proteins secretion [43]. Previous study reported that the Sec62/Sec63 protein complex plus BiP could promote Sec61 channel opening and thereby improving the secretion efficiency of proteins [44]. The PDI (M440DRAFT_1464388) were significantly upregulated at the translational level both in two mutants, which plays an important role in mediating protein folding and correct conformation forming in the ER [45], thereby enhancing the processing efficiency of enzyme protein in mutants due to the protein folding were regard as a rate-limiting step in protein production at sufficient nutrition supply [46].

The translation levels of VIP36 (M440DRAFT_1396316) protein involved in secretory pathway were significantly increased by 3.66 and 4.13-fold in LC-M4 and LC-M16 mutants when compared with the WT, respectively. This protein could interact with various cargo category in the transport vesicles (such as GPI anchors, glycoproteins, secretory protein or glycolipids) and aid in its sorting and transport to the cell surface [47], and thus improving the protein secretion efficiency in mutants. Therefore, these ER chaperones and folding enzymes associated with the protein secretory pathway are often the targets of overexpression for cellulase enzyme production via genetic engineering technique in filamentous fungi [48].

The glycosylation of enzyme protein, a structurally and widely diverse form of post-translational modification, is vital in localization, protein stability and secretion [49, 50]. The N-linked glycosylation of proteins has been extensively reported in the yeast [51] and mammalian systems [52]. The N-glycan biosynthesis pathway was comment shared by the DEGs and DEPs, which indicated that a universal glycosylation occurred during enzyme production in the hyper-producing mutants. In this study, the N-glycan biosynthesis-related genes, oligosaccharyltransferase (OST, M440DRAFT_359740) and mannosyl-oligosaccharide glucosidase (MOGS, M440DRAFT_1329096) were significantly up-regulated at the translational level even though its down-regulated at the transcriptional level. The OST is a multimeric protein complex located at the membrane of ER and catalyzes the glycosylation reaction, which affects the folding and the sorting of proteins in the ER [53]. Therefore, we speculated that the N-linked glycosylation modification may mediate the cellulolytic enzyme protein secretion in mutants and play an important role in the regulation enzyme synthesis in *T. longibrachiatum.* Similarly, Mathew et al., reported that the unusual glycosylation modifications on N-glycans of cellobiohydrolase I were detected in high cellulase-producing mutants of *T. reesei *[54]. In the present study, although the detailed molecular mechanism underlying the cellulase hyper-producing activity of LC-M4 and LC-M16 mutants remains unknown, the above results might be useful for the genetic engineering of *T. longibrachiatum* to improve cellulolytic enzyme production for use in bio-refinery applications.

## Conclusions

In the present study, the cellulase hyper‑producing mutants of LC-M4 and LC-M16 derived from the wild type *T. longibrachiatum* LC strain through heavy ion mutagenesis exhibited the growth phenotype variations and high-efficiency secretion ability of cellulase and hemicellulose. In addition, the integrative analysis of transcriptome and proteome showed that the protein processing in ER involved in protein secretory pathway, starch and sucrose metabolism pathway and N-glycan biosynthesis pathway were significantly changed both in cellulase hyper-producing mutants, which maybe the main reason of cellulase hyper-production in LC-M4 and LC-M16 mutants. Moreover, a hypothetical secretory model of cellulase protein in filamentous fungi was established on the basis of DEGs/DEPs and key candidate genes (PDI, Sec61, VIP36, OST, MOGS) identified from the omics analysis, which supplemented the process of cellulase metabolism regulation and synthesis secretion pathway. Taken together, the results of this study were of great guidance on the rational genetic engineering and/or breeding of filamentous fungi for improving cellulase production.

## Supplementary Information

**Additional file 1: Table S1.** Sequence of primers used for RT-qPCR.

**Additional file 2: Table S2.** Quality summary of RNA-Seq data.

**Additional file 3: Fig S1.** Volcano plots showing genes with differential expression in the LC-M4 vs LC group (A) and LC-M4 vs LC group (B) and proteins with differential expression in the LC-M4 vs LC group (C) and LC-M4 vs LC group (D), respectively.

**Additional file 4: Table S4.** Gene differentially expressed analysis and result of LC-M16_vs_LC.

**Additional file 5: Table S5.** Gene differentially expressed analysis results of LC-M4_vs_LC.

**Additional file 6: Fig. S2.** Comparison of the gene expression levels by RNA-seq and RT-qPCR.

**Additional file 7: Table S6.** Common shared DEPs and DEGs of LC-M4 vs LC.

**Additional file 8: Table S7.** common shared DEPs and DEGs of LC-M16 vs LC.

## Data Availability

The datasets used and/or analyzed during the current study are available from the corresponding author on reasonable request.
