# Supplementary material for: Integrative transcriptome and proteome analyses of Trichoderma longibrachiatum LC and its cellulase hyper-producing mutants generated by heavy ion mutagenesis reveal the key genes involved in cellulolytic enzymes regulation
Source: Biotechnol Biofuels Bioprod. 2022 Jun 3;15:63. doi: 10.1186/s13068-022-02161-7 (PMC9166314; doi:10.1186/s13068-022-02161-7)
Supplement: Supplementary file 1 — Additional file 1: Table S1. Sequence of primers used for RT-qPCR. [file 13068_2022_2161_MOESM1_ESM.docx]

Additional file 1: Table S1. Sequence of primers used for RT-qPCR.

| Gene ID | Sense Primer (5’-3’) | Anti-Sense Primer (5’-3’) |
| --- | --- | --- |
| M440DRAFT_1434316 | AGATGGCAGAGGAATAGC | TCTCCGTGCGATATGAC |
| M440DRAFT_1402014 | CTTGTAAGCCTCTTGATGTG | TATCCTGTGTTGCCGAATA |
| M440DRAFT_1394562 | CGCTCTACTATGGCTACAA | TGTTATCTCGGAGGTAGTTG |
| M440DRAFT_6177 | CTTGGGCGTATAGGTAGTT | CTGTTGATGATTGCTGGAT |
| M440DRAFT_5564 | ACGAGCACGGTATATGTTA | CATCAACTTAGAGACCTTACAC |
| M440DRAFT_1402167 | GACTTCAACTTCTTATACACTCA | GTATGAATGATAAGCAGATGGT |
| M440DRAFT_1405546 | GTATAGATTCAACTGCGGTATG | GGCTGGAGAATGATGATTAG |
| M440DRAFT_561 | TGAATGTTCTTGTAGGTGATG | CTCTCCCTCTTGTAAATACGA |
| M440DRAFT_1334905 | GTGATACGAAGGTAGAGTGA | CTCTTTGTCCCTCATACTACT |
| M440DRAFT_1402365 | GCGTGAAGGAGATTAGGAT | GATAGAATAAGCCAGTGAAGTC |
| M440DRAFT_1397114 | AATGTTATACTTACCTGCCTTG | GATGCTTCTGCCAGTTATG |
| M440DRAFT_64036 | TCGTCAAGAGCCTTAGTATC | GTGTCGAATGCAAGAGTAG |
| M440DRAFT_66627 | ATGTAAGTTACGAGCCATTG | CGGCAAGTCATACAGAATC |
| M440DRAFT_1343409 | CAGACCTCATTGACAGATTC | ACGGGTATATCTTGGACTTT |
| M440DRAFT_1158211 | GAGGAGTAAATGGGACAGT | CATCCAAGTTAGCAAGAGTAG |
| M440DRAFT_1328625 | GCTTACTCCAGACAACAAC | TATTAGTCGCTGCTTCCAT |
| M440DRAFT_1389492 | GAGTGACCAATGTAGATGTTG | CCTGATTGCTAACCTGTTG |
| M440DRAFT_1398220 | TGTGAAGACTGATGGTGAT | TGTATAAGAGAACGGTAATGTG |
