# Supplementary material for: Integrative transcriptome and proteome analyses of Trichoderma longibrachiatum LC and its cellulase hyper-producing mutants generated by heavy ion mutagenesis reveal the key genes involved in cellulolytic enzymes regulation
Source: Biotechnol Biofuels Bioprod. 2022 Jun 3;15:63. doi: 10.1186/s13068-022-02161-7 (PMC9166314; doi:10.1186/s13068-022-02161-7)
Supplement: Supplementary file 2 — Additional file 2: Table S2. Quality summary of RNA-Seq data. [file 13068_2022_2161_MOESM2_ESM.docx]

Table S2 Quality summary of RNA-seq data

| Sample  ID | Raw  reads | Clean  reads | Clean bases | GC(%) | Q20(%) | Q30(%) | Mapped reads (%) |
| --- | --- | --- | --- | --- | --- | --- | --- |
| LC-1 | 41878824 | 41203942 | 5.75G | 57.85 | 97.44 | 92.93 | 89.15 |
| LC-2 | 52143914 | 51503108 | 7.17G | 57.66 | 97.8 | 93.71 | 89.90 |
| LC-3 | 48392686 | 47707448 | 6.62G | 57.69 | 97.75 | 93.6 | 89.36 |
| LC-M4-1 | 44284410 | 43590698 | 6.05G | 57.56 | 97.23 | 92.48 | 89.44 |
| LC-M4-2 | 50497922 | 49778940 | 6.92G | 57.63 | 97.67 | 93.44 | 89.70 |
| LC-M4-3 | 44694524 | 44006514 | 6.12G | 57.83 | 97.69 | 93.48 | 89.67 |
| LC-M16-1 | 48190448 | 47532340 | 6.61G | 57.51 | 97.69 | 93.46 | 89.43 |
| LC-M16-2 | 43568996 | 42784450 | 5.95G | 57.62 | 97.32 | 92.72 | 88.75 |
| LC-M16-3 | 46798512 | 45911060 | 6.39G | 57.95 | 97.54 | 93.18 | 89.63 |
