# Supplementary figures and images for: Integrative transcriptome and proteome analyses of Trichoderma longibrachiatum LC and its cellulase hyper-producing mutants generated by heavy ion mutagenesis reveal the key genes involved in cellulolytic enzymes regulation
Source: Biotechnol Biofuels Bioprod. 2022 Jun 3;15:63. doi: 10.1186/s13068-022-02161-7 (PMC9166314; doi:10.1186/s13068-022-02161-7)

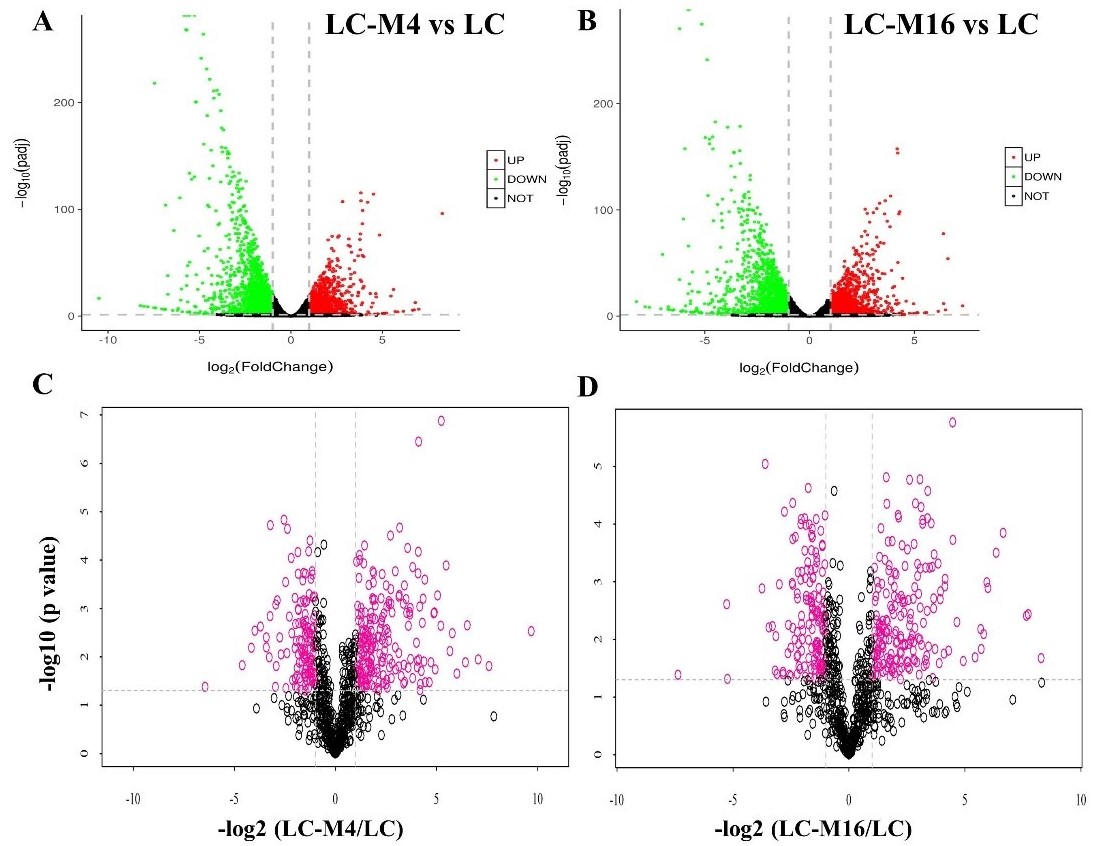

Supplement: Supplementary file 3 — Additional file 3: Fig S1. Volcano plots showing genes with differential expression in the LC-M4 vs LC group (A) and LC-M4 vs LC group (B) and proteins with differential expression in the LC-M4 vs LC group (C) and LC-M4 vs LC group (D), respectively. [file 13068_2022_2161_MOESM3_ESM.jpg]

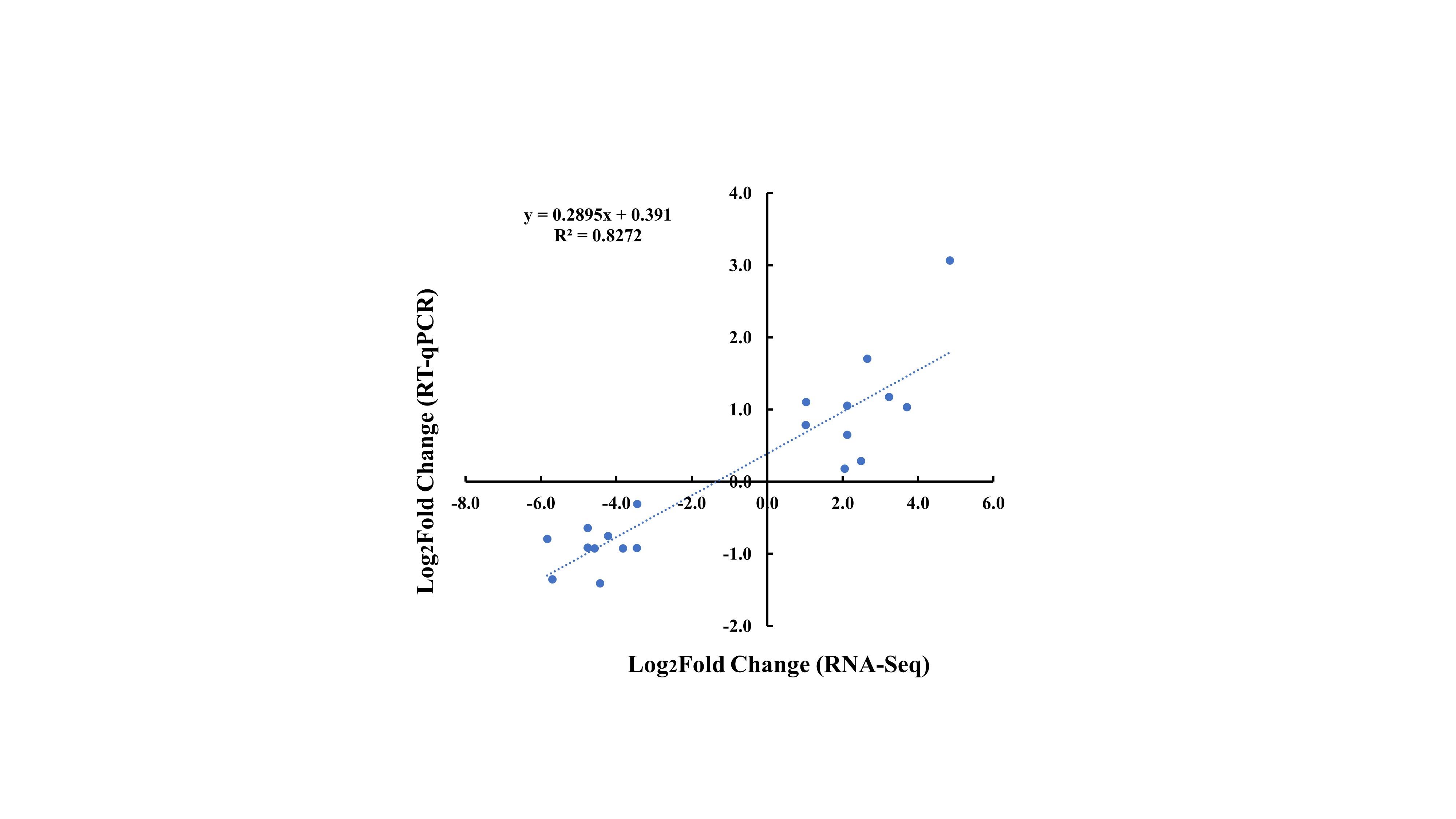

Supplement: Supplementary file 6 — Additional file 6: Fig. S2. Comparison of the gene expression levels by RNA-seq and RT-qPCR. [file 13068_2022_2161_MOESM6_ESM.jpg]
